# The 20S Proteasome Splicing Activity Discovered by SpliceMet

**DOI:** 10.1371/journal.pcbi.1000830

**Published:** 2010-06-24

**Authors:** Juliane Liepe, Michele Mishto, Kathrin Textoris-Taube, Katharina Janek, Christin Keller, Petra Henklein, Peter Michael Kloetzel, Alexey Zaikin

**Affiliations:** 1 Institut für Biochemie, Charité, Universitätsmedizin Berlin, Berlin, Germany; 2 Centre for Bioinformatics, Division of Molecular Biosciences, Imperial College London, London, United Kingdom; 3 Interdepartmental Center for Studies on Biophysics, Bioinformatics and Biocomplexity ‘L. Galvani’ (CIG), University of Bologna, Bologna, Italy; 4 Institute for Women's Health and Department of Mathematics, University College London, London, United Kingdom; Utrecht University, The Netherlands

## Abstract

The identification of proteasome-generated spliced peptides (PSP) revealed a new unpredicted activity of the major cellular protease. However, so far characterization of PSP was entirely dependent on the availability of patient-derived cytotoxic CD8+ T lymphocytes (CTL) thus preventing a systematic investigation of proteasome-catalyzed peptide splicing (PCPS). For an unrestricted PSP identification we here developed SpliceMet, combining the computer-based algorithm ProteaJ with *in vitro* proteasomal degradation assays and mass spectrometry. By applying SpliceMet for the analysis of proteasomal processing products of four different substrate polypeptides, derived from human tumor as well as viral antigens, we identified fifteen new spliced peptides generated by PCPS either by *cis* or from two separate substrate molecules, i.e., by *trans* splicing. Our data suggest that 20S proteasomes represent a molecular machine that, due to its catalytic and structural properties, facilitates the generation of spliced peptides, thereby providing a pool of qualitatively new peptides from which functionally relevant products may be selected.

## Author Summary

MHC class I molecules present antigenic peptides derived from endogenously expressed foreign or aberrant protein molecules to the outside world so that they can be specifically recognised by cytotoxic T lymphocytes (CTLs) at the cell surface. Responsible for the generation of these peptides is the 20S proteasome, which is the major proteolytic enzyme of the cell. These peptides were so far believed to exhibit a linear sequence identical to that found in the unprocessed parental protein. Using patient derived CTL it was previously shown that by proteasome catalyzed peptide splicing, i.e., by fusion of two proteasome generated peptide fragments in a reversed proteolysis reaction, novel spliced antigenic peptides can be generated. To resolve the CTL dependence of spliced-peptide identification we here performed experiments, which combined mass spectrometric analysis of proteasome generated peptides with a computer based algorithm that predicts the masses of all theoretically possible spliced peptides from a given substrate molecule (SpliceMet). Using this unrestricted approach we here identified several new spliced peptides of which some were derived from two distinct substrate molecules. Our data reveal that peptide splicing is an intrinsic additional catalytic property of the proteasome, which may provide a qualitatively new peptide pool for immune selection.

## Introduction

The multiple subunit 20S proteasome is the central catalytic unit of the ubiquitin proteasome system (UPS) and catalytic core of the 26S proteasome that is built by the association of the two 19S regulator complexes with the catalytic 20S core [19S-20S-19S]. With its N-terminal threonine residues as the single active site of the β-subunits β1, β2 and β5, the 20S proteasome is a N-terminal nucleophilic hydrolase responsible for the generation of the vast majority of virus- or tumor-derived peptides presented by MHC class I molecules at the cell surface for recognition by peptide-specific cytotoxic T lymphocytes (CTL) [1], [2]. This function is generally aided by the interferon-γ- (IFN-γ)-induced synthesis of the alternative catalytic subunits β1i, β2i, β5i, with concomitant formation of immunoproteasome subtypes possessing altered proteolytic properties as well as by the IFN-γ-induced up-regulation of the proteasome activator subunits PA28-α and PA28-β [3], [4]. Peptides generated by the 20S proteasome were so far thought to exhibit a linear sequence identical to that found in the unprocessed parental protein. This view was dramatically changed by the identification of three epitope peptides derived from the melanocyte protein gp100, the SP100 nuclear phosphoprotein and fibroblast growth factor (FGF-5), which represented fusions of proteasomal cleavage products and were shown to be generated by proteasomes [5]–[8]. Proteasome-catalyzed peptide splicing was proposed to be a transpeptidation reaction whereby the acylester intermediate is stabilized at the active site formed by the N-terminal threonine of the catalytic subunits for a time span that is sufficient to allow the N-termini of the released peptide fragments to make a nucleophilic attack on the ester bond of the acyl-enzyme intermediate thereby forming a new peptide bond and producing the spliced peptides [6], [9]. Under physiological conditions proteolysis is normally favoured over hydrolysis. Therefore the formation of new immunologically relevant MHC class I ligands by proteasome catalyzed peptide splicing (PCPS) was exciting and raised the possibility that reverse proteolysis may be functionally more frequent and important than previously thought. Nevertheless, as only three spliced epitope peptides had been reported in the literature since their initial discovery in 2004 it was assumed that PCPS might rather be a rare event. It was also emphasized, however, that presently available database search algorithms fail to detect peptide splicing products [10]. Moreover, the fact that identification of spliced peptides remained fortuitous due to the dependence on the accidental availability of patient derived CTLs so far prevented a systematic investigation of PSP.

It appears reasonable to assume that, similar to conventional proteasomal cleavage products, not every spliced peptide will fulfil the quality requirements of a MHC class I ligand. Thus, considering the generation of spliced antigenic peptides recognized by patient derived CTL, one might predict that the cellular proteasomal splicing reaction, as such, must be a considerably more frequent event than so far assumed. But even if peptide splicing is a rare event, PSP may still play a crucial role within the immune response. This is due to the sensitivity of CTL cells, which are able to detect very small numbers of MHC class I peptide complexes [11], and in the most extreme example even a single MHC class I complex [12].

**Figure 1:**
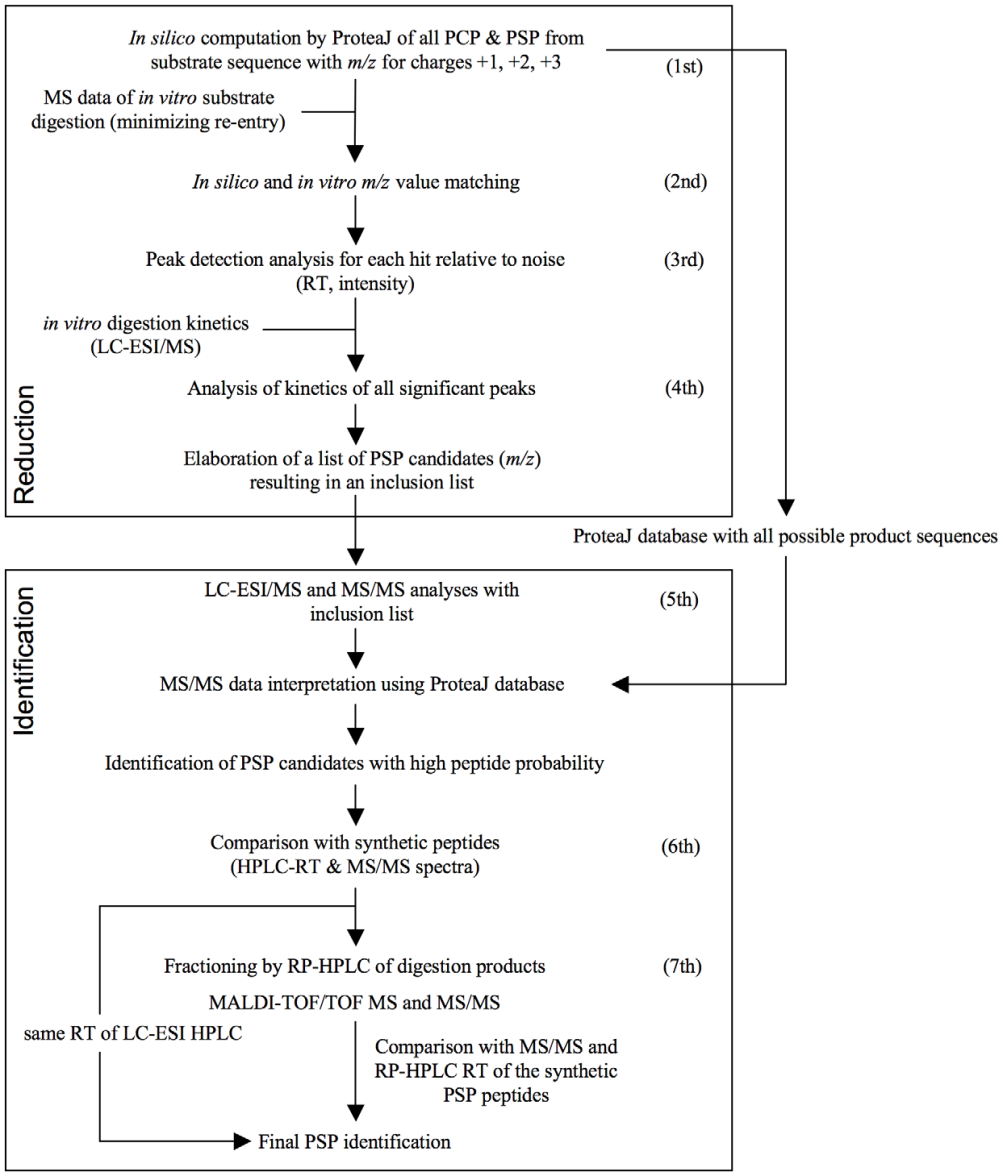
SpliceMet. Applying the computer program ProteaJ on a peptide sequence of choice, *m/z* values of all theoretically possible proteasomal cleavage (PCP) and splicing (PSP) products are calculated (1^st^ step). This is followed by an *in vitro* digest of the synthetic substrate and the comparison of the obtained MS signals with the theoretical *m/z* values (2nd). Matching of the signals and verification of peptide generation kinetics results in an inclusion list for LC-ESI-MS/MS analysis required for identification of the PSP (3rd, 4th). For final confirmation, the MS/MS spectra (5th) and the HPLC-RT of proposed PSP (6th) are compared with those of the analogous synthetic peptides. For the identification of those PSP candidates that do not fully satisfy these requisites, the generation of PSP is up-scaled followed by HPLC fractionation with an extended gradient and the fractions are analyzed by nano-LC-MALDI-TOF/TOF-MS (7th).

To allow a systematic, CTL-independent investigation of PSP we therefore developed SpliceMet: a method that combines combinatorial computations (ProteaJ) with mass spectrometric (MS) analyses of proteasome-generated peptides. Based on a given protein or peptide sequence, ProteaJ produces a data set with the *m/z* value of all theoretically possible PSP that may be generated by the proteasome through the combination of any two fragments (greater than one amino acid in length) generated from the same substrate molecule (in *cis*) or from separate substrate molecules (in *trans*) and ligated in a normal or reverse order. This is followed by MS analysis of *in vitro* digests of the synthetic peptide substrate and by comparison of the MS signals obtained with the theoretical ProteaJ-computed *m/z* values. By matching the theoretical values with the experimentally obtained *m/z* values and verifying the peptide generation kinetics, a restricted list of candidate PSP is generated. Their presence in 20S proteasome digests of substrates is then investigated by LC-ESI-MS/MS and LC-MALDI-TOF/TOF-MS/MS leading to the final identification of the PSP (Fig. 1).

## Results

### SpliceMet

The SpliceMet method is organized into two main experimental blocks characterized by 7 main steps (Figure 1). To reduce the number of possible proteasome generated spliced peptides (PSP) the first block utilizes the following 4 main steps that are subsequently investigated in the second block. The first experimental block combines the computational algorithm ProteaJ with proteasome *in vitro* digests of a synthetic peptide of choice and mass spectrometric (MS) analyses as follows:

#### 1) Calculation of all combinatorially possible PSP and setting of the ProteaJ database

The digestion of the substrate of length L with a sequence of amino acids a*_i_*, *i = 1..L* may result in 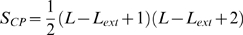 cleavage products (PCP) each of which can be denoted as PCP*_ij_*, where the product starts at the position *i, i = 1…L-L_ext_+1* (C-terminus) and ends at the position *j = i+L_ext_-1…L* (N-terminus). L_ext_ describes the minimal length of a PCP that can produce a PSP (here L_ext_  =  2). Any two PCP*_ij_* and PCP*_kn_* may be spliced into *PSP_i-j/k-n_*. For the total amount of generated products S_all_, including PCP and PSP, we have i = 1*…*L-L_ext_+1, j = i+L_ext_-1*…*L, k = 1*…*L-L_ext_+1, n = k+L_ext_-1*…*L and we can calculate 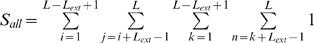. Note that 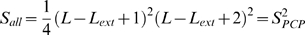. PSP can be classified into two main groups: *cis* splicing (PSP_cis_) and *trans* splicing (PSP_trans_), whereby *cis* splicing occurs in the same order as in the substrate (PSP_cis,normal_, where i+L_ext_≤j+1≤k+1≤n-L_ext_) or in reverse order (PSP_cis,reverse_, k+L_ext_≤n+1≤i≤j-L_ext_+1). The total number of all PSP is then 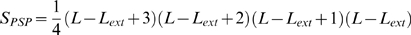. The number of pure *trans* PSP can be calculated as PSP_trans_ = S_PSP_-PSP_cis,normal_- PSP_cis,reverse_. Table 1 summarizes the conditions for each product and their total amount.

**Table 1:** Computation of cleavage and splicing products.

products	conditions	total amount
all fragments	i = 1*…*L-L_ext_+1, j = i+L_ext_-1*…*L, k = 1*…*L-L_ext_+1, n = k+L_ext_-1*…*L	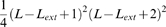
PCP	*i = 1…L-Lext+1 j = i+L_ext_-1…L*	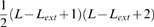
cis - normal	i = 1*…*L-2L_ext_ j = i+L_ext_-1*…*L-L_ext_-1 k = j+2*…*L-L_ext_+1 n = k+L_ext_-1*…*L	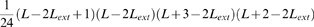
cis - reverse	k = 1*…*L-2L_ext_+1 n = k+L_ext_-1*…*L-L_ext_ i = n+1*…*L-L_ext_ j = i+L_ext_-1*…*L	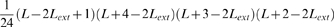

Described are the conditions to compute all products of a specific type (PCP, cis-normal PSP and –reverse PSP). The indices i, j, k and n are the amino acid positions of the product, *e.g.* PSP_i-j,k-n_, L is the length of the substrate, L_ext_ is the minimal length of a PCP that can produce a PSP.

#### Estimation of mass-to-charge ratios (m/z) of all possible PSP

To list all possible PSP, in which four indices *ijkn* define the sequence, we computed the molecular weight (Mr, calc.) of each peptide and the corresponding *m/z* values for charge states z = 1, 2, 3 (*m/z* =  (Mr+z)/z). Since the *m/z* values of PSP can differ by less than the mass accuracy of 0.5 Da for the used ESI-ion trap mass spectrometer (LCQ-classic & DECA XP instruments), we clustered all *m/z* values into groups with a *m/z* range of 0.2 Da (accordingly to the MS instrument resolution). For each group we determined the average *m/z* value thereby obtaining a set of theoretical *m/z* values that could be further analyzed.

#### 2) Matching with the LC-ESI/MS full spectra

The presence of the theoretical *m/z* values was detected among MS signals of the digestion products of the investigated peptide of choice.

#### 3) Peak detection of all the computed m/z values

In the LC-ESI mass chromatogram we identified the significant peaks for each theoretical *m/z* value. For each theoretical *m/z* value either no peak or several peaks could be detected and defined by their *m/z* and retention time (RT).

#### 4) Analysis of m/z time-dependent kinetics and establishment of an inclusion list for the LC-ESI/MS measurements

In time-dependent processing experiments (signal intensity versus time of digestion) identified peaks that did not fulfill the following criteria were eliminated from the candidate list: i. initial intensity (t = 0) smaller than MAX (*e.g.* here  = 10^7^ for measurements by DECA XP MAX instrument); ii. monotonously ascending signal intensity towards a maximum followed by a monotonous decline in case assay condition allowed re-entry of the PSP. It was assumed that the monotonous increase resulted from the continuous production of PSP and the decrease from the “re-entry” event.

Next, we defined t_max_ as the digestion time when the highest amount of generated PSP was observed and sorted all pairs (*m/z*, RT) with respect to t_max_ into groups indexed as g of the size D_g_. If D_g_>D_max_ (here 15 depending on MS resolution) then the corresponding group was split into subgroups g_i_ of size smaller than D_max_. The number of groups determined the number of additional up-scaled processing assays in which the absolute concentration of substrate and proteasome were increased keeping the relative substrate/proteasome ratio constant, whereas the total number of subgroups represented the number of requested new MS runs. The resulting *m/z*, RT, t_max_ established the inclusion list.

The second block consists of the following 3 steps:

#### 5) LC-ESI-MS/MS analysis with inclusion list

Precursor ion selection for MS/MS analysis was performed using the established inclusion list enabling the fragmentation analysis of even low-abundance peptides. MS/MS spectra were analyzed with Bioworks software version 3.3 (Thermo Fisher) using the ProteaJ database. Significant hits which were annotated as PSP showed a peptide probability p<0.00005.

#### 6) Comparison with synthetic peptides

All identified PSP resulting from step 5 were manually confirmed by comparison with synthetic peptides of the same sequence. The candidate PSP and their synthetic analogues had to exhibit a similar RT (delta RT <0.5 min) and fragmentation pattern in the LC–ESI-MS/MS analysis.

#### 7) Validation of PSP sequences by MALDI-TOF

In some experiments the requirements outlined in step 5 and 6 were not fully met requesting further MS identification. In this case, we proceeded by fractionating the digestion products by reverse phase (RP)-HPLC and by analyzing each fraction by LC-ESI-MS/MS using an inclusion list with the *m/z* values of the PSP candidates. Their RT in the HPLC run was also compared with that of the corresponding synthetic peptides. Those fractions with MS/MS and RT that matched the PSP were lyophilized and fractionated again using a more focused HPLC method to decrease the number of peptides in each fraction. The up-scaled fractions were subsequently compared with the RT of the synthetic PSP and analyzed by nano-LC-MALDI-TOF/TOF-MS/MS.

### Validation of SpliceMet

**Figure 2:**
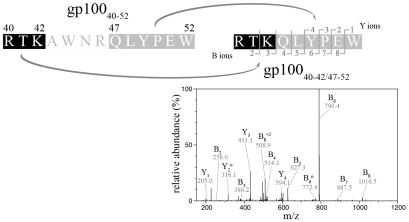
By applying SpliceMet we identified the known PSP produced by digestion of the synthetic 13mer gp100_40–52_ by 20S proteasomes. Sequence of the substrate gp100_40–52_ and of the PSP gp100_40–42/47–52_ and its ESI-MS/MS spectrum (double protonated with *m/z* 610.8) are shown. In the spectra B- and Y-ions are reported. Ions' loss of water is symbolized by °. In the experiments (100 µl of reaction) 4 nmol of gp100_40–52_ were cleaved for 36 hours by 1 µg 20S proteasome purified from LcL.

For proof of principle we initially investigated 20S proteasome catalyzed peptide splicing during proteasomal degradation of the synthetic 13mer peptide (gp100^PMEL17^_40–52_, RTKAWNRQLYPEW), previously shown to serve as substrate for PSP generation [6]. For the experiments we used 20S proteasomes of Lymphoblastoid cell Lines (LcL), which possess splicing activity [7] and predominantly resemble the immunoproteasome subtype [13], [14]. Following each step of SpliceMet we obtained a progressive decrease of the number of candidate PSP leading to the identification of the previously described PSP gp100^PMEL17^_40–42/47–52_ [6] by LC-ESI/MS/MS at the 6^th^ step of SpliceMet (Figure 2). The substantial reduction of PSP in the candidate list (Table 2) and the final identification of the PSP gp100^PMEL17^_40–42/47–52_ validated our analysis method.

**Table 2:** PSP candidate reduction by applying SpliceMet.

	number of m/z				number of sequences		
SpliceMet steps	1	2	3	4	5	6	7
**gp100_40–52_**	2580 (100)	280 (10.8)	32 (1.2)	18 (0.7)	2	1	
**gp100_35–57_**	7229 (100)	1288 (17.8)	1121 (15.5)	239 (3.3)	20	4	5

Reduction of number of PSP candidates during the progression of SpliceMet step by step. The number of possible PSP detectable in the *in vitro* digestion of a peptide declines continuously during the consecutive steps of SpliceMet (Figure 1). Here the PSP number reduction observed for the 13mer gp100_40–52_ and 23mer gp100_35–57_ is reported both as total number and as a percentage compared to the theoretical PSP number (in brackets). The values are referring to the number of possible PSP at the end of the SpliceMet step. For example, although 5664 PSP could be generated from gp100_40–52_ assuming 2 as the minimum length of the native PCP (L_ext_), only 2580 represent the *m/z* value clusters (obtained with a cluster range of 0.2) that will be matched with the LC-ESI/MS full spectrum at the beginning of step 2. Moreover, up to step 4 the numbers are referred to as the number of *m/z* values whereas from step 5 they are referred to as the possible sequence because they have been identified by MS/MS.

**Figure 3:**
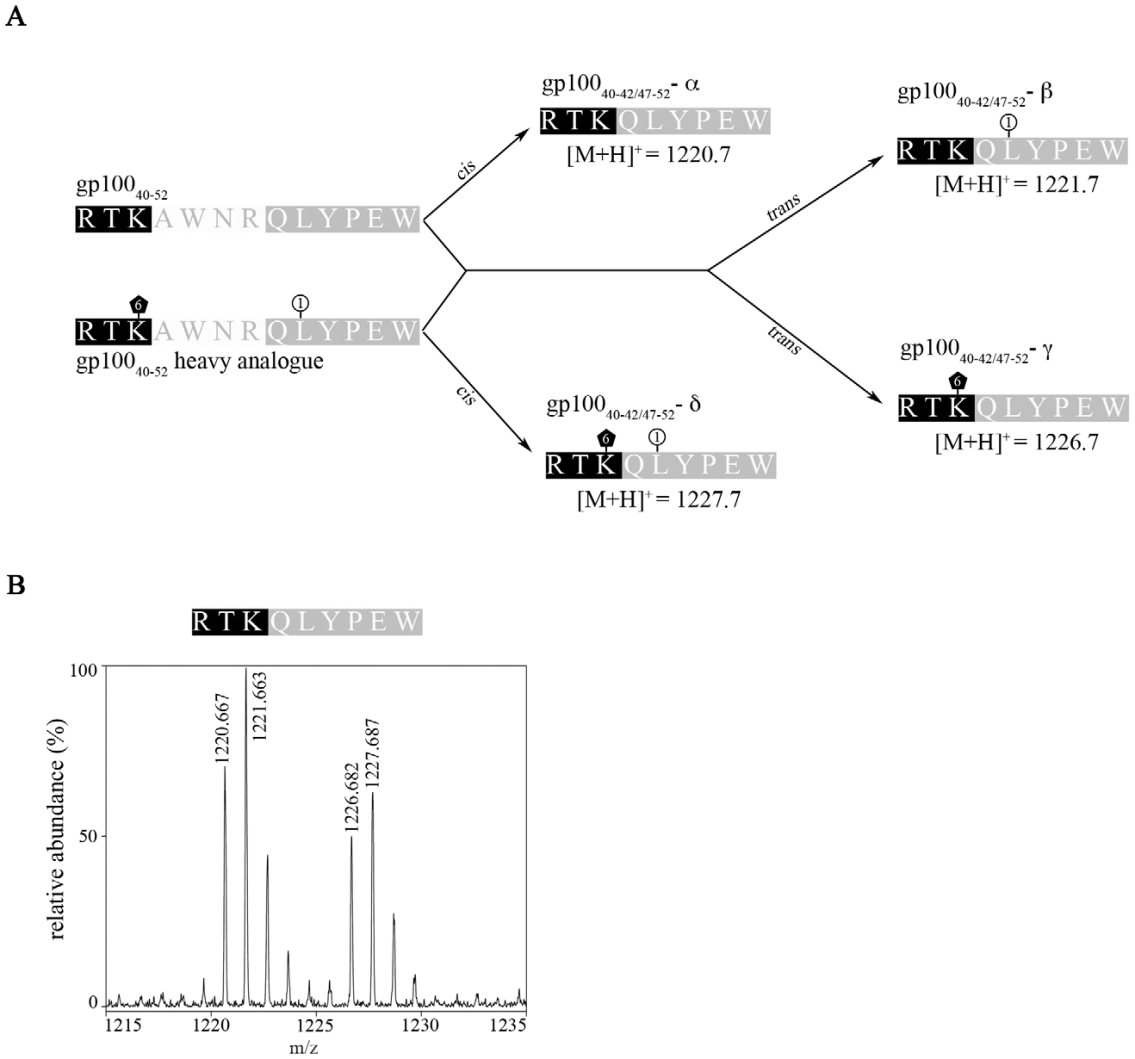
Generation of PSP by proteasomal *trans* splicing. (**A**) To demonstrate the generation of a PSP*_trans_* by the binding of two fragments originated from two distinct molecules of substrate, 5 nmol gp100_40–52_ and its heavy analogue with amino acids ^13^C_6_-Lys and ^15^N-Leu (RTK^+6^AWNRQL^+1^YPEW) were digested together for 36 hours by 1.5 µg LcL 20S proteasomes in 100 µl buffer. Theoretically four different PSP could be generated from the *cis* or *trans* ligation of the proteasomal fragments [RTK] and [QLYPEW] with sequences [RTK][QLYPEW]: gp100_40–42/47–52_-α, [M+H]^+^  = 1220.7; gp100_40–42/47–52_-β, [M+H]^+^  = 1221.7; gp100_40–42/47–52_-γ, [M+H]^+^  = 1226.7; gp100_40–42/47–52_-δ, [M+H]^+^  = 1227.7. (**B**) LC-MALDI-TOF/TOF-MS spectra at RT  = 41.3 min show peaks which can be assigned to all four possible PSP of gp100_40–42/47–52_ (for MS/MS spectra see Figure S1).

To verify the hypothesis of the occurrence of a proteasome-dependent *trans* splicing reaction we performed *in vitro* digestions in which the unmodified 13mer gp100_40–52_ peptide was applied to proteasomal processing in the presence of the same peptide but with the heavy amino acid residues ^13^C_6_-Lys and ^15^N-Leu (RTK^+6^AWNRQL^+1^YPEW). As shown in Figure 3, we indeed detected PSP variants as being the results of *cis* (variants −α & −δ) or of *trans* (variants −β & −γ) splicing, demonstrating that PCPS can occur not only in *cis* but also in *trans* (see also Figure S1).

### Identification of nine new PSP in the proteasomal digestion of gp100_35–57_

**Figure 4:**
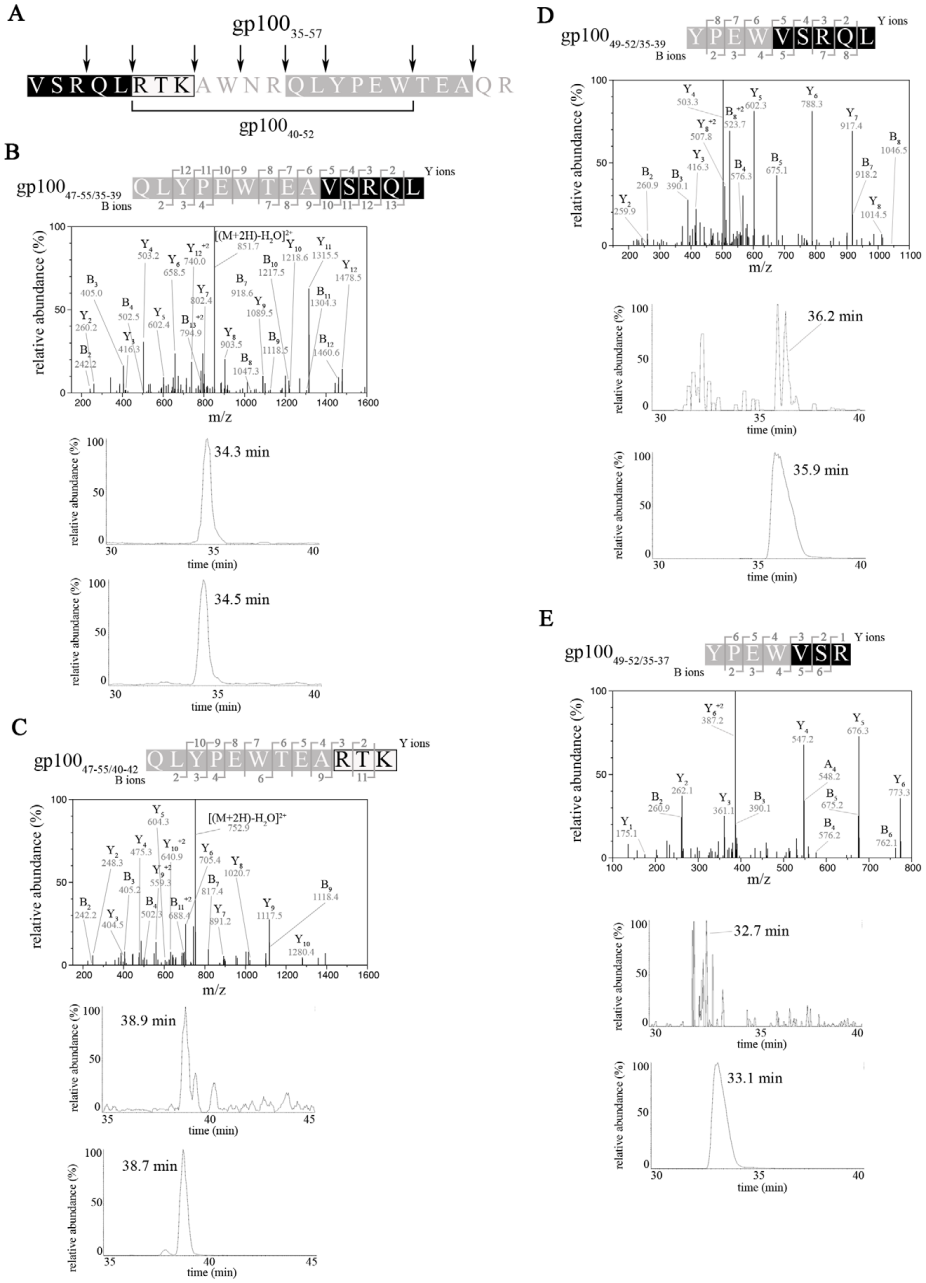
Identification of PSP in gp100_35–57_ digestion by SpliceMet [step 6]. (**A**) Sequence of gp100_35–57_. The bracket indicates the previously described substrate gp100_40–52_. Arrows indicate the cleavage positions that are necessary to generate the newly identified PSP. The colors correspond to the identified PSP sequences as reported in (**B**) to (**E**). (**B–E**) LC-ESI/MS/MS spectra (upper panels) and extracted ion chromatograms (middle and lower panels) of the double-protonated PSP. (**B**) [QLYPEWTEA][VSRQL] (gp100_47–55/35–39_) - *m/z* 860.4, (**C**) [QLYPEWTEA][RTK] (gp100_47–55/40–42_) - *m/z* 761.6, (**D**) [YPEW][VSRQL] (gp100_49–52/35–39_) – *m/z* 589.5, (**E**) [YPEW][VSR] (gp100_49–52/35–37_) – *m/z* 469.0. The RT of the detected peaks in the digestion is consistent (with a maximum difference of 0.5 min) with those of synthetic peptide of same sequences (lower panel of extracted ion chromatograms). 40 µM gp100_35–57_ were digested for 24 hours in 100 µl reaction by 0.5 µg 20S proteasomes purified from LcLs.

**Figure 5:**
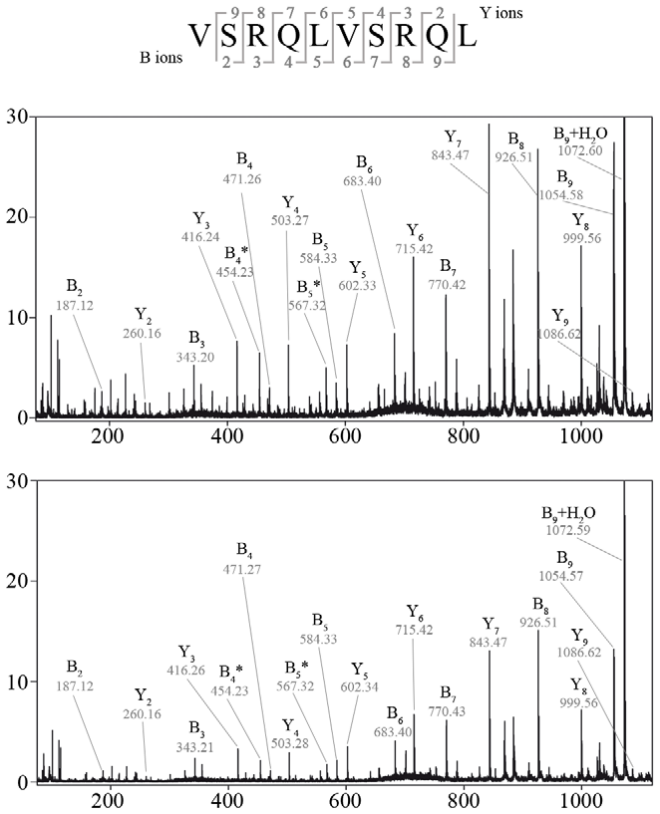
MS/MS identification of the PSP_trans_ gp100_35–39/35–39_ [VSRQL][VSRQL] within the gp100_35–57_ digestion products. MALDI-TOF/TOF-MS/MS spectrum of precursor ion [M+H]^+^  = 1185.7 observed in the up-scaled digestion (see “Methods” section) of the 23mer gp100_35–57_ by 20S LcL proteasomes (upper panel) is shown in comparison with the synthetic peptide [VSRQL][VSRQL] (lower panel). In the spectra B-, Y-ions, the loss of ammonia symbolized by *, the relative abundance (%) in y-axis and *m/z* in the x-axis. are reported.

By applying SpliceMet we investigated the generation of new PSP derived from the proteasomal degradation products of the 23mer peptide gp100_35–57_, which is a N- and C- terminally extended version of gp100_40–52_ by LcL 20S proteasome (Figure 4A). In these experiments we identified eight new PSP*_cis_*, four of which were identified at step 6 (Figure 4) and four at step 7 of SpliceMet (Table 3 & Figure S2). We also identified a ninth PSP with the sequence [VSRQL][VSRQL] derived from splicing of two distinct molecules of the PCP gp100_35–39_ (Figure 5). The identification of this PSP was of particular relevance because it was the first example of PSP*_trans_* detected in *in vitro* proteasomal digestion of a single peptide sequence.

**Table 3:** PSP identified in the proteasomal digestion of the polypeptide gp100_35–57_.

Peptide (gp100)	Sequence	Mr, calc	PSP type	Identification step of SpliceMet
49–52/35–39	[YPEW][VSRQL]	1176.59	*cis,reverse*	6
49–50/35–37	[YPEW][VSR]	935.45	*cis,reverse*	6
47–55/40–42	[QLYPEWTEA][RTK]	1520.76	*cis,reverse*	6
47–55/35–39	[QLYPEWTEA][VSRQL]	1718.86	*cis,reverse*	6
47–52/35–37	[QLYPEW][VSR]	1176.59	*cis,reverse*	7
47–48/35–39	[QL][VSRQL]	842.50	*cis,reverse*	7
45–52/35–37	[NRQLYPEW][VSR]	1446.74	*cis,reverse*	7
37–38/49–57	[RQ][YPEWTEAQR]	1462.70	*cis,normal*	7
35–39/35–39	[VSRQL] [VSRQL]	1184.70	*trans*	7

The PSP identified by the application of SpliceMet on the proteasome-mediated digestion of the substrate gp100_35–57_ are here described. PSP_normal_ or PSP_reverse_ result from splicing in the same order as the substrate or in reverse order to the substrate, respectively. PSP*_cis_* are derived from the splicing of two non-overlapping sequences of the original substrate. In contrast, PSP*_trans_* necessarily originate from two distinct substrate molecules because of the overlapping sequences of the two peptides spliced together.

### PSP formation is a general phenomenon not restricted to the gp100_35–57_ sequence

Since the sequence requirement for PCPS are not yet known one might argue that the observed frequent PSP generation when gp100^PMEL17^_35–57_ was used as substrate was due certain gp100_35–57_ sequence specificities. To test this we applied SpliceMet for the analysis of PSP derived from another polypeptide sequence of the same protein, *i.e.* gp100_201–229_. Among the proteasome-generated degradation products of this 29mer we identified three PSP (Table 4 and Figure S3). Since peptide fragments with overlapping sequences were spliced together these PSP were generated by a *trans* splicing event.

**Table 4:** PSP identified in proteasomal digestions of three additional polypeptides.

PSP	Sequence	Mr, calc	PSP type	Identification step of SpliceMet
***substrate* gp100_201–230_- AHSSSAFTITDQVPFSVSVSQLRALDGGNK**				
201–204/201–209	[AHSS][AHSSSAFTI]	1301.60	*trans*	6
201–209/201–207	[AHSSSAFTI][AHSSSAF]	1607.70	*Trans*	7
201–207/201–207	[AHSSSAF][AHSSSAF]	1393.61	*trans*	7
***substrate* gag-pol_29–58_*-* YKLKHIVWASRELERFAVNPGLLEVTSEGC**				
45–57/48–49	[AVNPGLLEVTSEG][PG]	1439.58	*trans*	6
***substrate* pp89_16–40_*–* RLMYDMYPHFMPTNLGPSEKRVWMS**				
27–30/23–30	[PTNL][PHFMPTNL]	1381.61	*trans*	6
27–32/20–30	[PTNLGP][DMYPHFMPTNL]	1943.89	*trans*	6

To verify that the relatively high PSP number was not peculiar to the sequence gp100_35–57_ we extended our investigation to three additional peptides. Six new PSP were identified within their products of *in vitro* proteasomal digest by applying SpliceMet. Three of them derived from the digestion of the sequence gp100_201–230_, one from HIV gag-pol_29–58_ and two from MCMV pp89_16–40_. All of them were produced by a *trans* splicing reaction.

In order to exclude a peculiar and rare tendency of the entire gp100 sequence to be spliced by PCPS we investigated the *in vitro* digestion products of two other peptides, *i.e.* the 30mer HIV-derived gag-pol_29–58_ and the murine cytolomegalovirus (MCMV)-derived 25mer polypeptide pp89_16–40_. The *in vitro* processing of gag-pol_29–58_ by proteasomes produced at least one PSP*_trans_* (Table 4 & Figure S4), whereas two PSP*_trans_* were detected after the digestion of the MCMV derived pp89 polypeptide peptide (Table 4 & Figure S5).

## Discussion

### SpliceMet

The aim of our study was to develop a method for the identification of spliced peptides which would allow the identification of any theoretically possible PSP and which was independent of adventitiously available CD8+ T cells and T-cell recognition assays permitting the detection of only a single spliced epitope peptide. The availability of such a method would greatly facilitate systematic studies required to elucidate the molecular mechanism of PCPS. Therefore we have developed and applied a method – SpliceMet – that, by combining computational and experimental methods, facilitates the identification of proteasome-generated spliced peptides.

Although in this investigation we have considered only polypeptide substrates up to a length of 30 amino acid residues, SpliceMet could also be applied to longer peptides or proteins to further our understanding of the mechanisms that govern PCPS and, in particular, *trans*-splicing. It has to be pointed out however that an increase in substrate length will lead to an exponential expansion of the ProteaJ data base as well as the number of peaks detectable by MS and therefore will require the application of restricting parameters such as size or sequence quality to match this approach with the capacity of the presently available MS technologies.

In our experiments we observed a substantial number of peak spectra at the 5^th^ step of SpliceMet, which could not be identified with sufficient confidence due to the low MS/MS quality. The number of unidentified spectra depends on the size of the ProteaJ database and to technical difficulties of MS analysis. Therefore, to reduce the number of unidentifiable spectra we incorporated the 7^th^ step into our method. Indeed, up-scaling of the digestion products by two rounds of HPLC fractionation permitted a better separation of the digestion products thereby limiting the number of overlapping peptides with similar *m/z* and RT and increased product concentration in this manner facilitating the identification of PSP by MS. Furthermore, at step 7 we analyzed the sample with a second MS instrument, a MALDI-TOF/TOF mass spectrometer, which has a higher resolution and sensitivity than the used ESI-ion trap mass spectrometer. Its application in other studies allowed the identification of peptides not previously detected by ESI-MS/MS, not only because of the higher sensitivity but also due to the different method of ionization and detection, which led to the identification of a complementary pool of peptides [15], [16]. Accordingly, we used both techniques to identify as many PSP as possible. LC-ESI/MS analysis was primarily adopted because it is a less time consuming technique and allowed the analyses of as large a number of samples as needed at SpliceMet step 4. Likely, a further minimization of unidentified spectra could be obtained by exploiting the high performance of the new generations of MS analyzers.

The computational algorithm ProteaJ is based on a combinatorial approach. Therefore the amount of calculated PSP strongly depends on parameters like substrate length L and the minimal length of a PCP L_ext_, as well as the kind of PSP allowed, *i.e. cis* or *trans* PSP. Thus ProteaJ parameter settings were used which in preliminary experiments seemed to be most reliable; for example, we limited the PCP L_ext_ to a minimum of 2 and accordingly we identified PSP such as gp100_47–48/35–39_ or gag-pol_45–57/48–49_. In contrast, when we considered PCP L_ext_  = 1 in a preliminary experiment on gp100_35–57_ we were not able to identify any new PSP (data not shown).

### SpliceMet applications and PSP implications

By applying SpliceMet we here showed that 20S proteasomes possess a substantial *in vitro* splicing activity. Since *in vitro* experiments for generation of spliced and non-spliced epitope peptides are known to closely resemble the *in vivo* situation [3] our data reveal that 20S proteasomes represent a molecular machine that facilitates the generation of spliced peptides from its own cleavage products. Therefore, our data may have considerable biological implications in that they provide evidence that proteasome-dependent protein degradation results in the generation of a second, so far undetected pool of spliced peptides, from which novel potentially functionally relevant peptides can be selected. Indeed, the two previously identified PSP were shown to be MHC class I epitopes recognized by CTL of human patients [6], [7]. This and the relatively high number of PSP that we identified raises the possibility that peptide splicing in general may lead to an increase in the peptide pool available for epitope selection. For example, from the melanocytic gp100^PMEL17^ tumor antigen (661 amino acids) 1,786,862 9mers with a unique sequence could be theoretically produced. Of these, a maximum of 652 are unspliced proteasomal cleavage products while the rest (99.96%) represent theoretical PSP. At the moment we do not have any sufficient information to judge on how many of these PSP (as well as normal PCP) are really produced and which percentage of them may efficiently bind MHC class I molecules. Based on our preliminary data we are tempted to speculate that specific PCP are generated more efficiently than PSP even if the MS signal of some PSP (*e.g.* gp100_47–55/35–39_) was as high as that of many PCP (data not shown). Nevertheless, if, for example PCP were produced 1000-fold more efficiently than any given PSP, spliced peptides generated from gp100^PMEL17^ would still represent a significant peptide pool (*i.e.* the 73.26% of the 9mers derived from the digestion of gp100^PMEL17^) from which antigenic spliced peptides could be selected.

This basic computational analysis assumes that the splicing of proteasomal cleavage products can occur also *in vivo*. Our observation that the *in vitro* splicing reaction not only occurs in *cis* but also in *trans* indirectly supports such an assumption. The existence of the *trans* PSP implies the likely situation that two or more substrate molecules are present at the same time within the proteasomal cavity as suggested by some excellent previous studies [17]–[19] or that the cleavage products of a first substrate molecule remain within the catalytic chamber while a second molecule of substrate is cleaved. Very recently, Dalet and co-workers investigated *trans* proteasome splicing *in vivo*, providing some very interesting albeit not final insights. They showed that PSP*_trans_* were generated *in vivo* when the precursor peptides of FGF-5 and gp100 were electroporated into COS cells, whereas only the FGF-5-derived PSP*_trans_* (and in a very small amount) could be detected by CTL assay when COS cells were transfected with FGF-5 or gp100 plasmid [8]. Taking into account the high number of PSP*_trans_* we identified within *in vitro* digestion products of four peptides, we are led to conclude that further studies *in vitro* and *in vivo* on different cellular and proteasome models are required to clarify this phenomenon.

An extensive application of SpliceMet on a wide range of polypeptides substrates would also help to identify putative peptide sequence motifs that facilitate the proteasomal splicing reaction. For example, in seven of the nine gp100_35–57_-derived PSP, the sequence VSR represents the N-terminus of those PCP, which according to the transpeptidation model [6], [20] perform a nucleophilic attack on the acyl-enzyme intermediate, thereby forming the detected PSP. Likewise, for four PSP the sequence YPEW represents the C-terminus, which forms the acyl-enzyme intermediate that is subsequently attacked by the second PCP generating the new PSP. From these observations one might infer a higher affinity of these two peptide sequences for a transpeptidation reaction. However, only a more extensive investigation of this specific issue with SpliceMet, covering a large number of different polypeptides would allow to validate such a hypothesis.

For this and other aims, studies performed with the help of SpliceMet could be powered if coupled with algorithms for the prediction of proteasomal cleavages, mathematical modeling of degradation kinetics as well as of the MHC class I antigen presentation [21]–[26]. Such an approach would also facilitate the reduction of the theoretical PSP number, which might represent a limitation of SpliceMet application to very long proteins such as gp100^PMEL17^. By combining the SpliceMet results with the estimation of these and other algorithms it would be theoretically possible to restrict the PSP identification to a group of PSP possessing features of interest (*e.g.* epitope-specific for a defined HLA I haplotype) and to predict their altered expression upon proteasome modification [24].

## Methods

### I. Peptides and peptide synthesis

All peptides were synthesized using Fmoc solid phase chemistry as previously described [27]. Exception had to be made for heavy analogues of gp100_40–52_. The isotope-labeled amino acids ^15^N- Fmoc-L-Leucine (3eq. amino acid, 3eq. HBTU, 6eq. DIEA in DMF) and L-Lysine-α-N-Fmoc, ε-N-T-Boc, ^13^C_6_ (1.92eq. amino acid, 1.92eq. HBTU, 3.84eq. DIEA in DMF) were coupled over night. The sequence enumeration for the peptides gp100_40–52_, gp100_35–57_ and gp100_201–229_ is referred to the human gp100^PMEL17^ sequence described by Adema and colleagues [28], for the peptide pp89_16–40_ is referred to the murine cytomegalovirus pp89 protein described by Lyons *et al.* [29]. The peptide sequence here named gag-pol_29–58_ is a modified version of the sequence 29–57 of the HIV gap-pol protein as described by Reitz *et al.* [30], where a Valin was inserted before the Threonin 53. All peptide sequences were extrapolated on the web site http://www.uniprot.org/.

### II. Cell cultures

Lymphoblastoid cell lines (LcLs) are human B lymphocytes immortalized with Epstein Barr virus (EBV) which mainly express active immunoproteasomes [13], [14]. LcLs were cultured in RPMI1640 medium supplemented with 10% FCS.

### III. 20S proteasome purification

20S proteasomes were purified from 3*E+09 LcLs as previously reported [31]. The purity of 20S proteasome preparation was verified by SDS-PAGE electrophoresis (12, 5% poly-acrylamide gel stained with Coomassie dye) (Figure S6). Furthermore, a non-proteasome proteolytic activity of the preparation was tested and excluded (data not shown) by the digestion of 40 µM gp100_40–52_ for 24 hours by 1 µg of LcL 20S proteasomes in presence of 400 µM Lactacystin (previously incubated with 20S proteasomes at room temperature for 10 min).

### IV. *In vitro* digestion of synthetic peptide substrates

Synthetic peptides at different concentrations (from 40 to 100 µM) were digested by 0.25–1.5 µg 20S proteasomes in 50–100 µl Hepes buffer (Hepes 20 mM, KCl 1 mM, MgCl 0.5 mM, DTT 1 mM, NaN_3_ 1 mM, pH 7.3) for different time periods (from 20 min to 48 hours) at 37°C. Digestions were stopped by acidic inactivation and frozen. Digestions were performed also in TEAD buffer (Tris 20 mM, EDTA 1 mM, NaN_3_ 1 mM, DTT 1 mM, pH 7.2) and no remarkable differences compared to Hepes buffer emerged (data not shown). In contrast, for SpliceMet step 7, 1.1 µmol of the peptides (at the final concentration of 100 µM) were digested for 24 hours by 62 µg of LcL 20S proteasomes in 10 ml Hepes buffer and the products up-scaled by RP-HPLC separation. All experiments reported in this study were repeated at least twice and each set of experiments was measured by each MS instrument at least twice.

### V. LC-ESI MS

In LC-runs the peptide separation was carried out on a 2.1 mm (µRPC C2/C18, 100 mm×2.1 mm, 3 µm, 120 Å, Amersham) and a 1 mm RP column (Beta Basic-18, 100 mm×1 mm, 3 µm, 150 Å, ThermoFisher) using a Surveyor system (ThermoFisher Scientific, USA). The mobile phase (A) was 100% water containing 0.05% (v/v) TFA and (B) was 70∶30 (v/v) acetonitrile/water containing 0.045% (v/v) TFA or 0.1% acetic acid for the PSP identifications reported in Figure 3. Online MS analysis was performed by DECA XP MAX iontrap instrument (ThermoFisher Scientific, USA) and by LCQ-classic iontrap (ThermoFisher Scientific, USA) after HPLC separation (HP1100, Agilent). MS data were acquired with a triple scan method in positive ion mode (MS - mass range 250–2000 *m/z*, zoom scan, MS/MS). Analysis of ESI/MS data was accomplished using Bioworks version 3.3 (ThermoFisher Scientific, USA). Database searching was performed using the ProteaJ database and the following parameters: no enzyme, mass tolerance for fragment ions 1amu. In time-dependent processing experiments (signal intensity versus time of digestion) we analyzed the kinetics of the identified peaks by using LCQuan software version 2.5 (Thermo Fisher). At step 3 of SpliceMet the significant peaks for each theoretical *m/z* value in the LC-ESI mass chromatogram were identified by Bioworks peak detection algorithm with a signal-to-noise ratio larger than δ (here  = 2).

### VI. Digestion product up-scaling by RP-HPLC

Further identification of the PSP at step 7 of SpliceMet was performed by MALDI-TOF/TOF-MS analysis of the gp100_35–57_ digestion products separated by two distinct rounds of RP-HPLC. In the first round 57 fractions were collected, lyophilized and analyzed by LC-ESI/MS to identify PSP candidates. The fractions containing the PSP candidates were then separated with more focused gradients (different for each selected fraction of the first round of HPLC separation) on the same column obtaining 47 fractions, which were lyophilized and investigated by MALDI-TOF/TOF-MS analysis. Each round was obtained by collecting the eluted fractions of the 5-15 runs (5–20 µl each) to maintain a good separation of the digestion products on the chromatogram. The runs were carried out on the column C18 (33×4.6 mm; ODS1 1.5 µm) by the HPLC Beckman SytemGold and different gradients of acetonitrile.

### VII. Nano-LC-MALDI-TOF/TOF-MS

Peptide separation was carried out using an Ultimate HPLC system (Dionex, Idstein, Germany). Samples were concentrated on a trap column (PepMap C18, 5 mm×300 µm×5 µm, 100 Å, Dionex) and eluted onto an analytical column (PepMap C18, 150 mm×75 µm×3 µm, 100 Å, Dionex). The mobile phase (A) was 2∶98 (v/v) acetonitrile/water containing 0.05% (v/v) TFA and (B) was 80∶20 (v/v) acetonitrile/water containing 0.045% (v/v) TFA. Runs were performed at a flow rate of 200 nL/min using a binary gradient 0–15% B in 4 min, 15–60% B in 45 min, 60–100% B in 5 min. Column effluent was mixed with MALDI matrix (5 mg/ml α-cyano-4-hydroxy-cinnamic acid in 70∶30 (v/v) acetontrile/water containing 0.1% (v/v) TFA, 1 µl/min) and spotted at ten second intervals on MALDI steel targets using a Probot fractionation device (Dionex). MS analysis was performed on a 4700 Proteomics Analyzer (Applied Biosystems, Framingham, MA, USA). MS data were acquired in positive ion mode in the mass range 800–4000 *m/z* by accumulation of 1200 laser shots per spot and processed with default calibration. MS/MS spectra were generated by 1 keV collisions and accumulation of 2500 to 10000 laser shots. Analysis of MALDI MS data was accomplished using MASCOT version 2.1 (Matrixscince, London, UK). Database search was performed using ProteaJ database and the following parameters: no enzyme, mass tolerance for precursors, +/− 80 ppm and for MS/MS fragment ions, +/− 0.3 Da. Spectral images for manual validation were prepared with Data Explorer Software version 4.8 (Applied Biosystems).

## Supporting Information

Figure S1 Verification of the PSP gp100_40–42/47–52_ with sequence RTKQLYPEW generated by *cis* and *trans* splicing.
(0.97 MB TIF)

Figure S2 MS/MS identification of four gp100_35–57_ PSP at step 7 of SpliceMet.
(0.55 MB TIF)

Figure S3 Identification of three PSP originated from the synthetic substrate gp100_201–230_.
(0.90 MB TIF)

Figure S4 Identification of the PSP gag-pol_45–57/48–49_.
(0.34 MB TIF)

Figure S5 Identification of two PSP originated from the synthetic substrate pp89_16–40_.
(0.63 MB TIF)

Figure S6 SDS-PAGE Electrophoresis with 20S proteasome purified from LcLs.
(0.95 MB TIF)
