# Supplementary figures and images for: The 20S Proteasome Splicing Activity Discovered by SpliceMet
Source: PLoS Comput Biol. 2010 Jun 24;6(6):e1000830. doi: 10.1371/journal.pcbi.1000830 (PMC2891702; doi:10.1371/journal.pcbi.1000830)

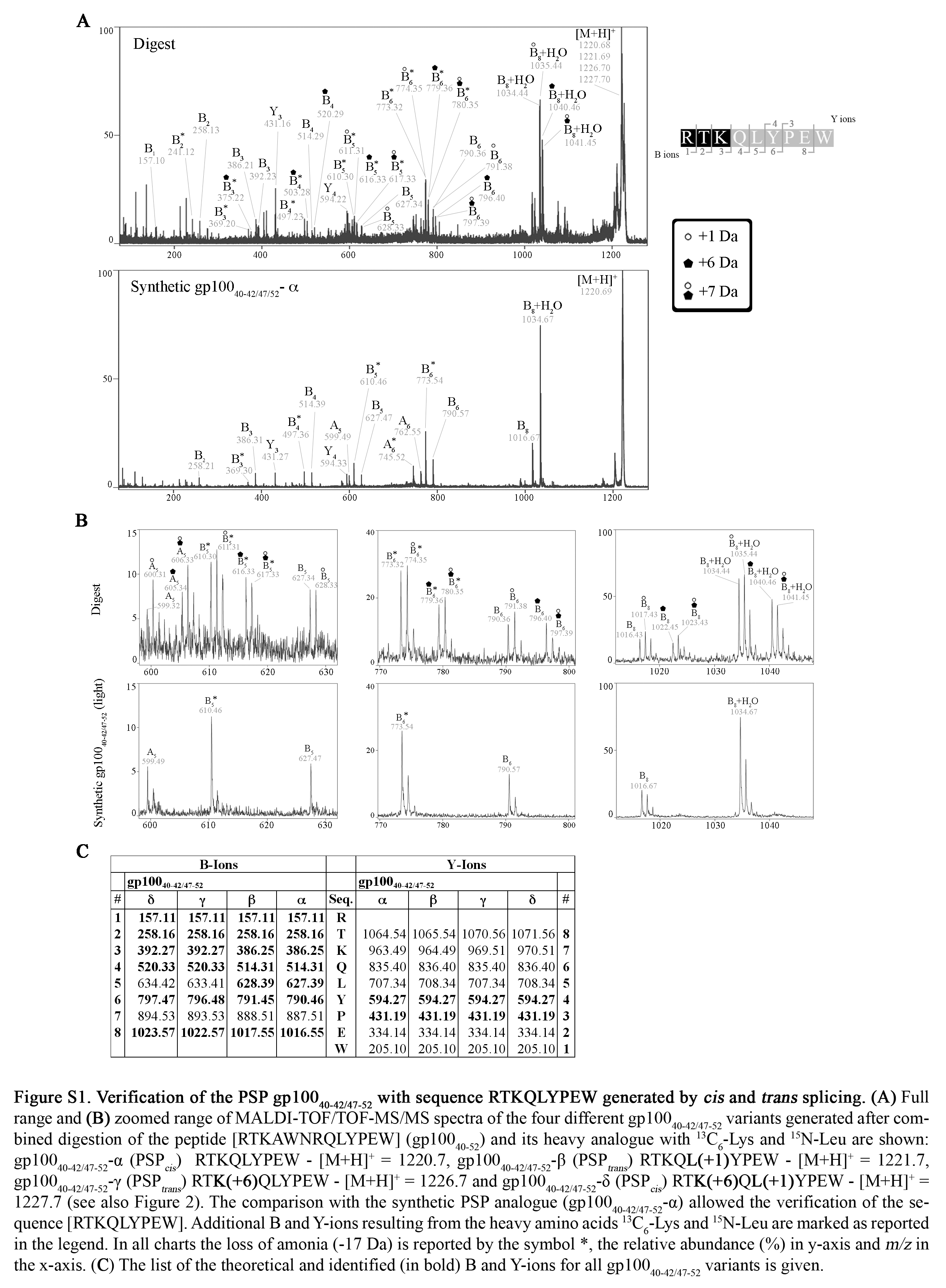

Supplement: Figure S1 — Verification of the PSP gp10040–42/47–52 with sequence RTKQLYPEW generated by cis and trans splicing. (0.97 MB TIF) [file pcbi.1000830.s001.tif]

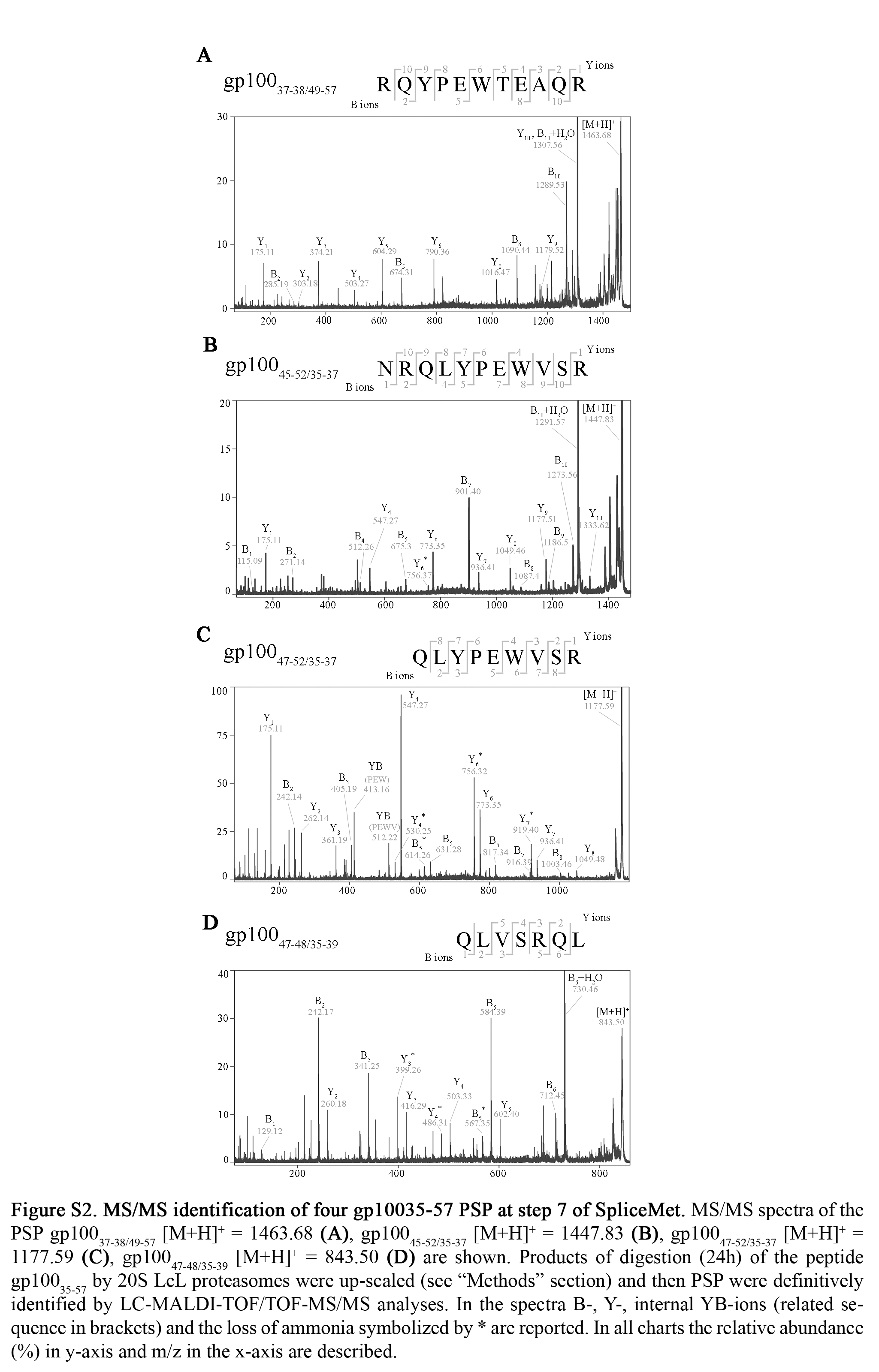

Supplement: Figure S2 — MS/MS identification of four gp10035–57 PSP at step 7 of SpliceMet. (0.55 MB TIF) [file pcbi.1000830.s002.tif]

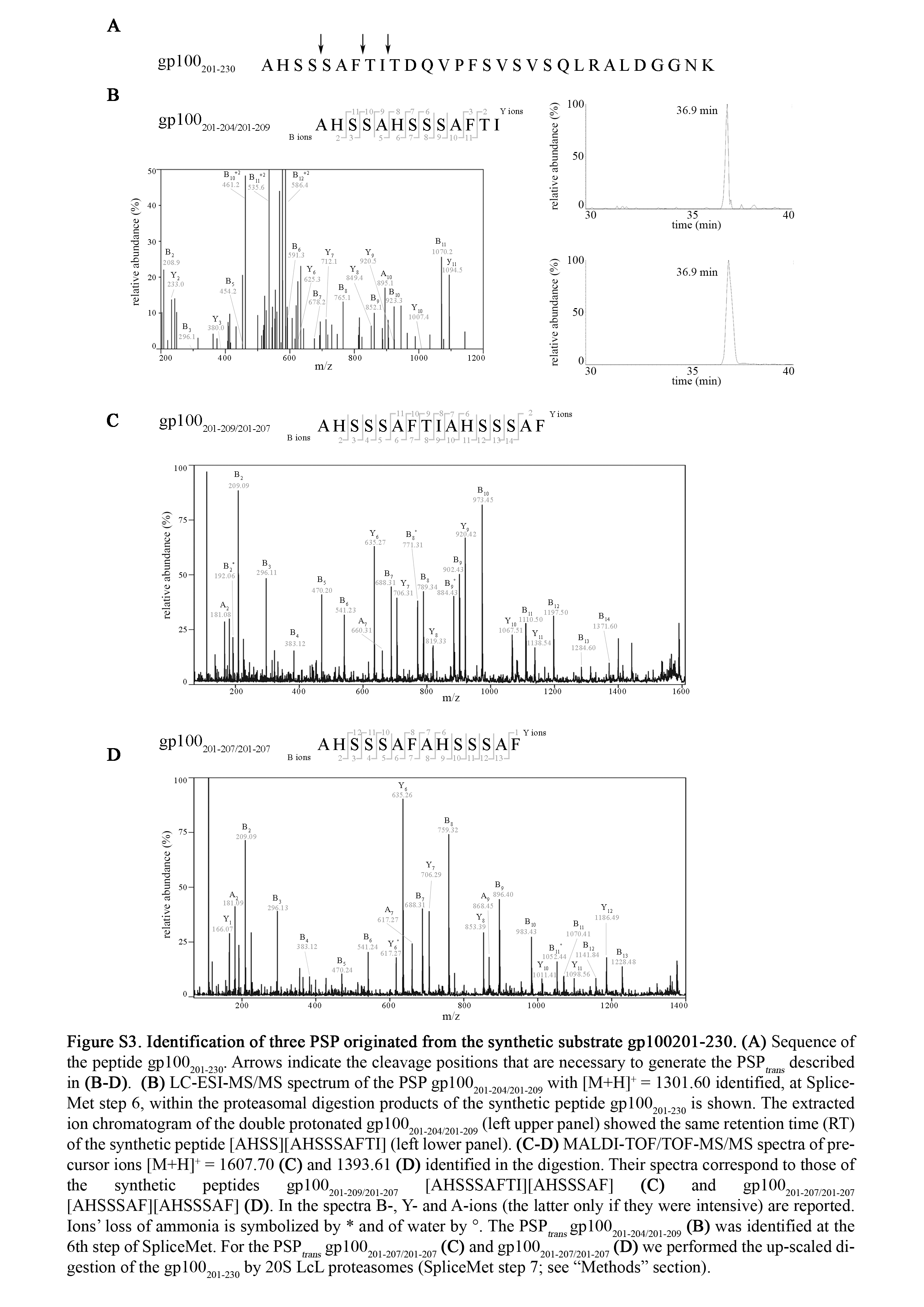

Supplement: Figure S3 — Identification of three PSP originated from the synthetic substrate gp100201–230. (0.90 MB TIF) [file pcbi.1000830.s003.tif]

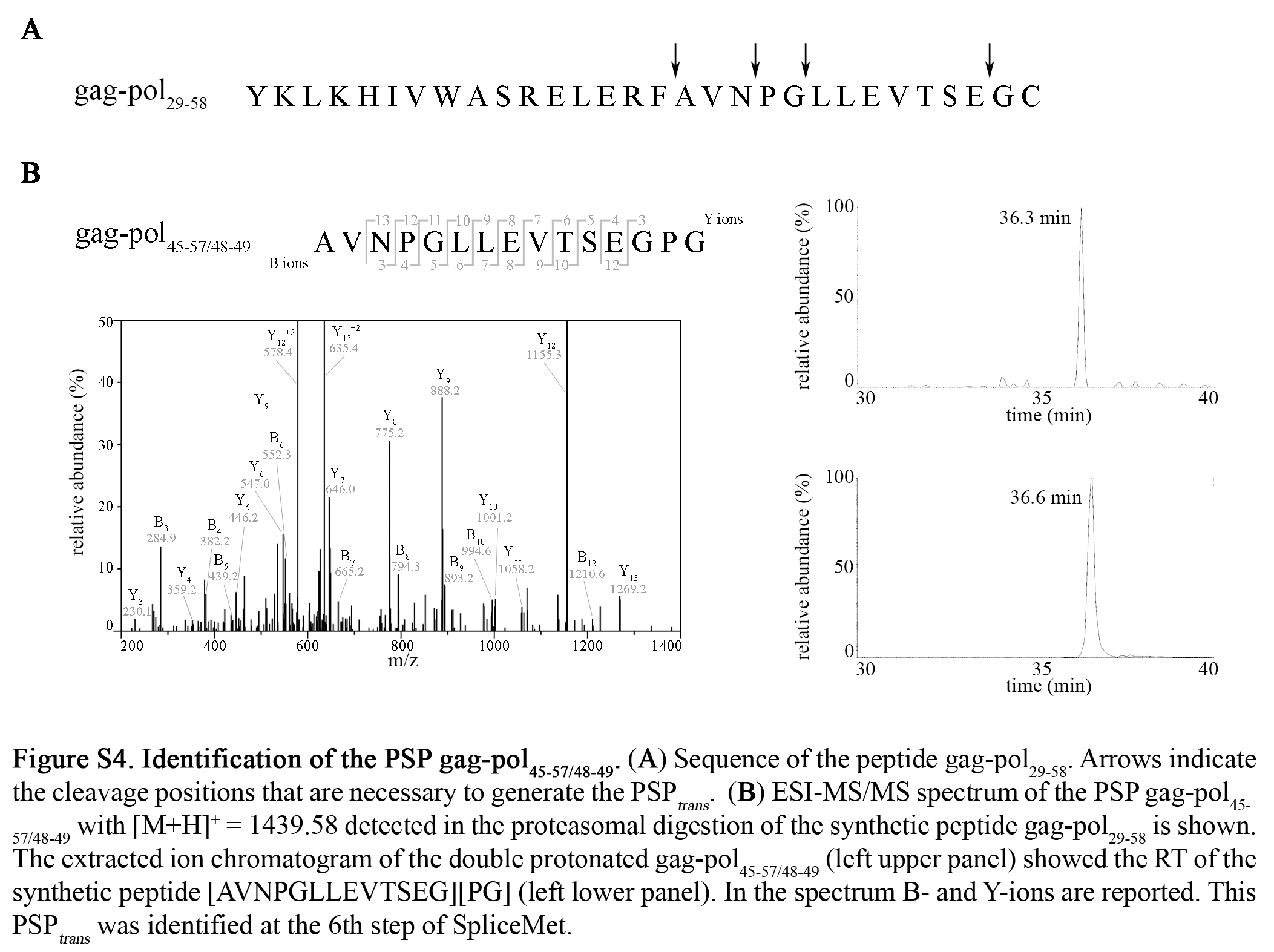

Supplement: Figure S4 — Identification of the PSP gag-pol45–57/48–49. (0.34 MB TIF) [file pcbi.1000830.s004.tif]

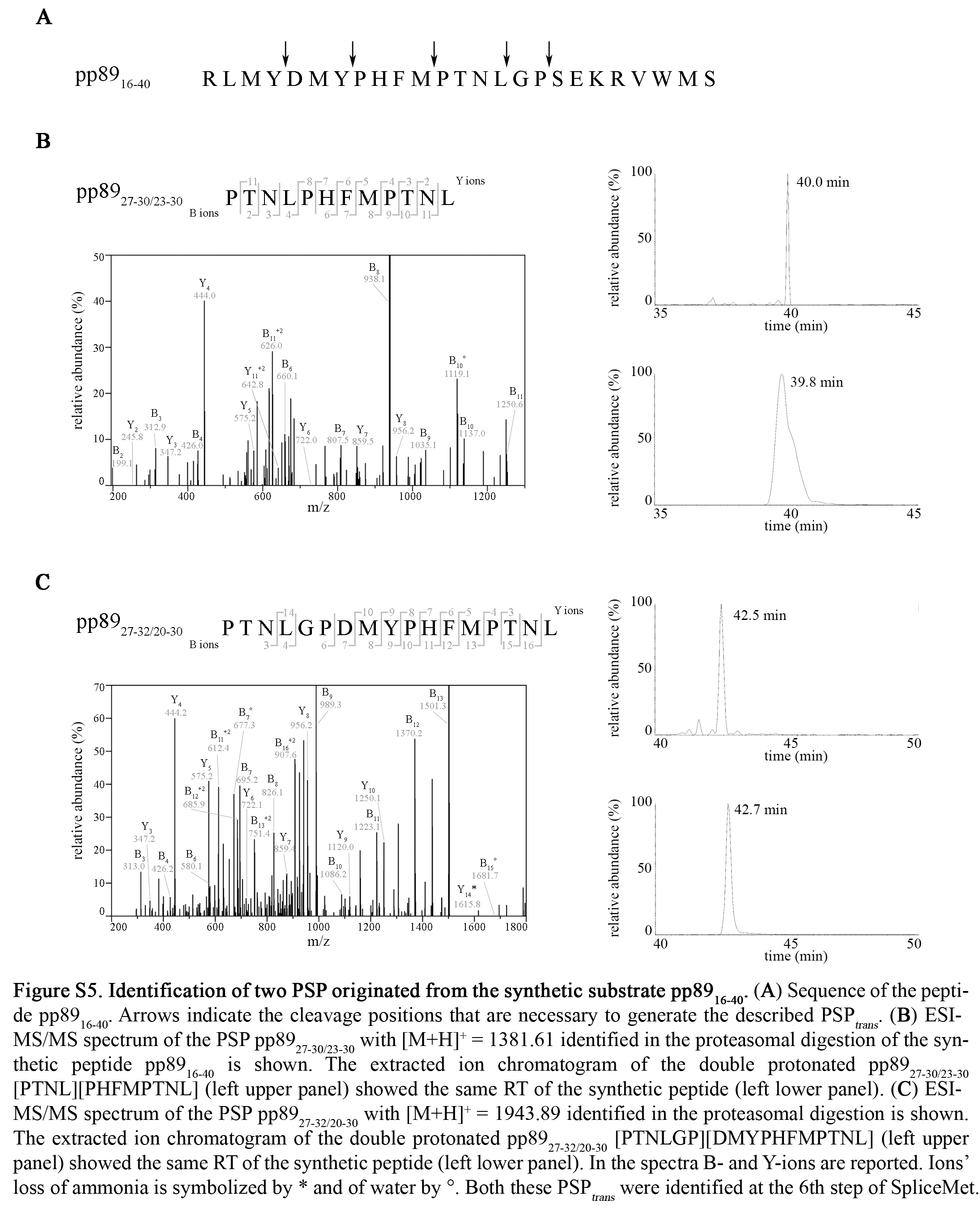

Supplement: Figure S5 — Identification of two PSP originated from the synthetic substrate pp8916–40. (0.63 MB TIF) [file pcbi.1000830.s005.tif]

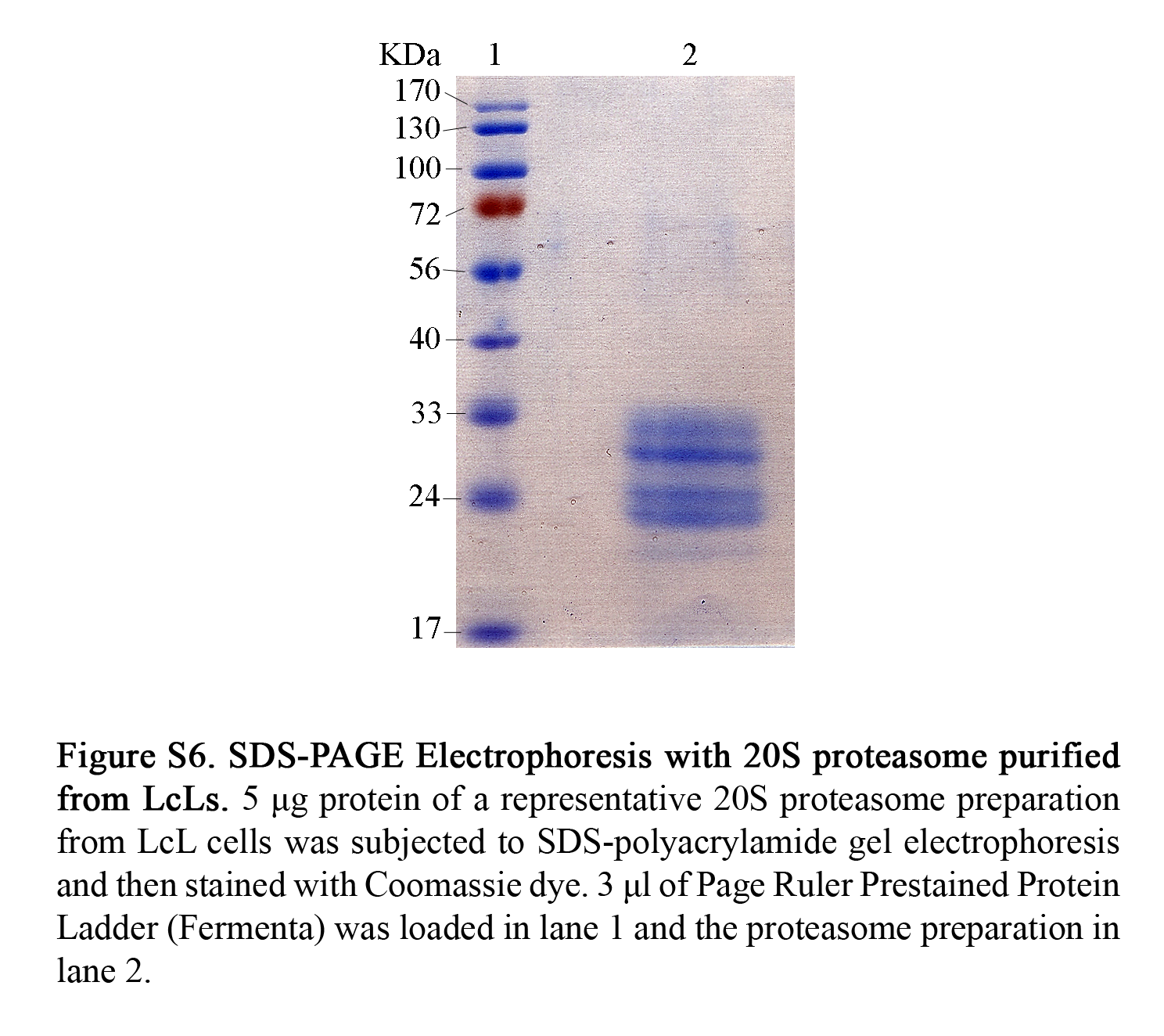

Supplement: Figure S6 — SDS-PAGE Electrophoresis with 20S proteasome purified from LcLs. (0.95 MB TIF) [file pcbi.1000830.s006.tif]
